# Fungal mitochondria govern both gliotoxin biosynthesis and self-protection

**DOI:** 10.1128/mbio.02401-25

**Published:** 2025-09-12

**Authors:** Patrícia Alves de Castro, Endrews Delbaje, Ivan Lucas de Freitas Migliorini, Monica T. Pupo, Muhammad Shafiul Alam Mondal, Karin Steffen, Antonis Rokas, Stephen K. Dolan, Gustavo H. Goldman

**Affiliations:** 1 Faculdade de Ciências Farmacêuticas de Ribeirão Preto, Universidade de São Paulo 67782 , Ribeirão Preto, Brazil; 2 Department of Genetics and Biochemistry, Eukaryotic Pathogens Innovation Center, Clemson University 2545, Clemson, South Carolina, USA; 3 Department of Biological Sciences, Vanderbilt University 5718, Nashville, Tennessee, USA; 4 Evolutionary Studies Initiative, Vanderbilt University 5718, Nashville, Tennessee, USA; 5 National Institute of Science and Technology in Human Pathogenic Fungi, São Paulo, Brazil; Institut Pasteur, Paris, France

**Keywords:** *Aspergillus fumigatus*, gliotoxin, transcriptional profiling, ubiquinone

## Abstract

Gliotoxin (GT) is a potent epipolythiodioxopiperazine toxin produced by the opportunistic pathogen *Aspergillus fumigatus* that contributes to virulence and inhibits competing microorganisms. However, GT is highly toxic to the producer itself, necessitating robust self-protection mechanisms. Here, we used a comparative transcriptomics approach between *A. fumigatus* (GT producer) and *A. nidulans* (non-producer) to identify additional genetic determinants of GT self-protection downstream of the transcription factor RglT. We characterized five RglT-dependent genes: *abcC1* (ABC transporter), *mfsD* (major facilitator superfamily transporter), *oxrA* (oxidoreductase), *mtrA* (putative methyltransferase), and *nmrC* (a GATA-type repressor). Deletion mutants in *A. fumigatus* and *A. nidulans* revealed that all except *oxrA* were required for full GT protection, with Δ*mtrA* and Δ*nmrC* exhibiting distinct phenotypes in oxidative stress and iron-starvation conditions. Transcriptomic profiling and protein network analysis showed that MtrA and NmrC influence mitochondrial functions, particularly ubiquinone biosynthesis, despite not localizing to mitochondria. Functional assays confirmed that GT exposure disrupts mitochondrial integrity and sensitizes *A. fumigatus* to mitochondrial inhibitors. Notably, GT-induced cell death was associated with mitochondrial fragmentation but lacked hallmarks of apoptosis-like nuclear damage. Together, our findings reveal new genetic components of GT detoxification and establish a critical role for mitochondrial function in *A. fumigatus* GT self-protection and production.

**IMPORTANCE:**

Gliotoxin (GT) plays a central role in the pathogenicity of *Aspergillus fumigatus* by enabling immune evasion and microbial competition, but its extreme toxicity also threatens the fungus itself. Although core GT biosynthetic and detoxification mechanisms are well studied, the full genetic network safeguarding against GT’s effects remains incompletely understood. This study identifies new RglT-regulated genes that contribute to GT self-protection and demonstrates that mitochondrial function is crucial for surviving GT exposure. Remarkably, similar protective pathways are active in both GT-producing and non-producing fungi, underscoring the ecological relevance of GT defense mechanisms. These findings deepen our understanding of fungal toxin tolerance and highlight mitochondria as a potential vulnerability that could be exploited for antifungal interventions.

## INTRODUCTION

*Aspergillus fumigatus* is the primary etiological agent of a group of heterogeneous diseases named aspergillosis, whose most lethal form is invasive pulmonary aspergillosis (IPA) (1). The predominance of *A. fumigatus* as a human pathogen is dependent on several biological attributes, among them the production of secondary metabolites (SMs). Fungal SMs can play the following roles: (i) as signaling molecules that mediate the communication of the fungus with its surroundings, (ii) as virulence factors to support pathogenic lifestyles, and (iii) as microbial inhibitors that shape the competition with other microorganisms for finite resources (2). One of these SMs is gliotoxin (GT), a sulfur-containing secondary metabolite that belongs to a class of naturally occurring 2,5-diketopiperazines produced by fungi (3, 4). GT is one of the most studied fungal secondary metabolites, and it is essential for *A. fumigatus* virulence and pathogenesis in humans and animals (3, 4). GT has a multitarget mechanism of action; it is fungicidal and bacteriostatic, induces apoptosis in mammalian cells, and modulates phagocytosis and neutrophil attraction (3, 4).

Interestingly, GT is also toxic against GT-producer and non-producer organisms (5). Very sophisticated mechanisms of GT self-protection have evolved in producers; some of these protective mechanisms are also found in non-producer organisms (5, 6). *A. fumigatus* GT biosynthetic gene cluster (BGC) has 13 genes located on chromosome 6, whose functions are defined for most of them. *A. fumigatus* GT self-protection is mainly based on the dual function of GliT, an oxidoreductase that functions as both a GT biosynthetic enzyme and a “protection” enzyme (7, 8) and the methyltransferase GtmA, whose gene is not in the GT BGC but in chromosome 2 (9). GliT enzymatically reduces oxidized GT to its dithiol form (dtGT) and GtmA methylates dtGT to form bisdethiobis(methylthio)-gliotoxin (bmGT), sequestering the reduced toxin. GtmA conversion to bmGT attenuates GT production post-biosynthetically (9–12). Both *gliT* and *gtmA* genes are not under the control of the transcription factor (TF) GliZ (3) but under the control of the TF RglT (5).

Fungal mitochondria play a central role in cellular adaptation to environmental stress by regulating energy metabolism, redox balance, and signaling pathways essential for survival under hostile conditions (13). Mitochondrial function is intimately linked to resistance mechanisms against antifungal drugs, particularly azoles and echinocandins, as mitochondrial activity affects ergosterol biosynthesis and cell wall integrity (4, 14, 15). For example, mutations in mitochondrial DNA or disruptions in mitochondrial function can lead to altered susceptibility to fluconazole in *Candida albicans*, suggesting that mitochondria contribute to drug tolerance and resistance development (16). Moreover, mitochondrial ROS production has been implicated in signaling pathways that induce adaptive responses during antifungal treatment, further underscoring their role in fungal persistence and pathogenicity (17).

The GT-nonproducer *A. nidulans* contains homologs of both *rglT* and *gliT* (but not *gtmA*). In *A. nidulans*, a Δ*rglT* mutant is highly sensitive to GT and cannot induce *gliT* expression (5). Previously, we used the strategy of comparing differential gene expression between GT non-producers and producers, aiming to identify additional genetic determinants involved in GT protection by comparing the *A. fumigatus* and *A. nidulans* corresponding wild-types and Δ*rglT* mutants exposed or not to sub-inhibitory GT concentrations (6). RNA sequencing reveals a markedly distinct transcriptional response to exogenous GT, involving the RglT-dependent regulon, which also exhibits significant differences between *A. fumigatus* and *A. nidulans*. However, we were able to observe 43 RglT-independent and 11 RglT-dependent homologs whose expression pattern was similar in both species. We identified a novel RglT-dependent methyltransferase, MtrA, involved in GT protection, and several transporters and at least one additional TF that are involved in GT protection and production. Here, we characterize the function *of A. fumigatus* and *A. nidulans* RglT-dependent genes *mtrA* (encoding a putative methyltransferase), *nmrC* (encoding a GATA-type transcription factor repressor), *mfsD* (major facilitator superfamily transporter), *abcC1* (ABC transporter), and *oxrA* (putative oxidoreductase). We also show that mitochondrial function is essential for GT production and self-protection, highlighting the roles of MtrA and NmrC in this process.

## RESULTS

### Molecular characterization of genes regulated by the transcription factor RglT in the GT-producing *A. fumigatus* and the non-GT-producing *A. nidulans*

**Fig 1:**
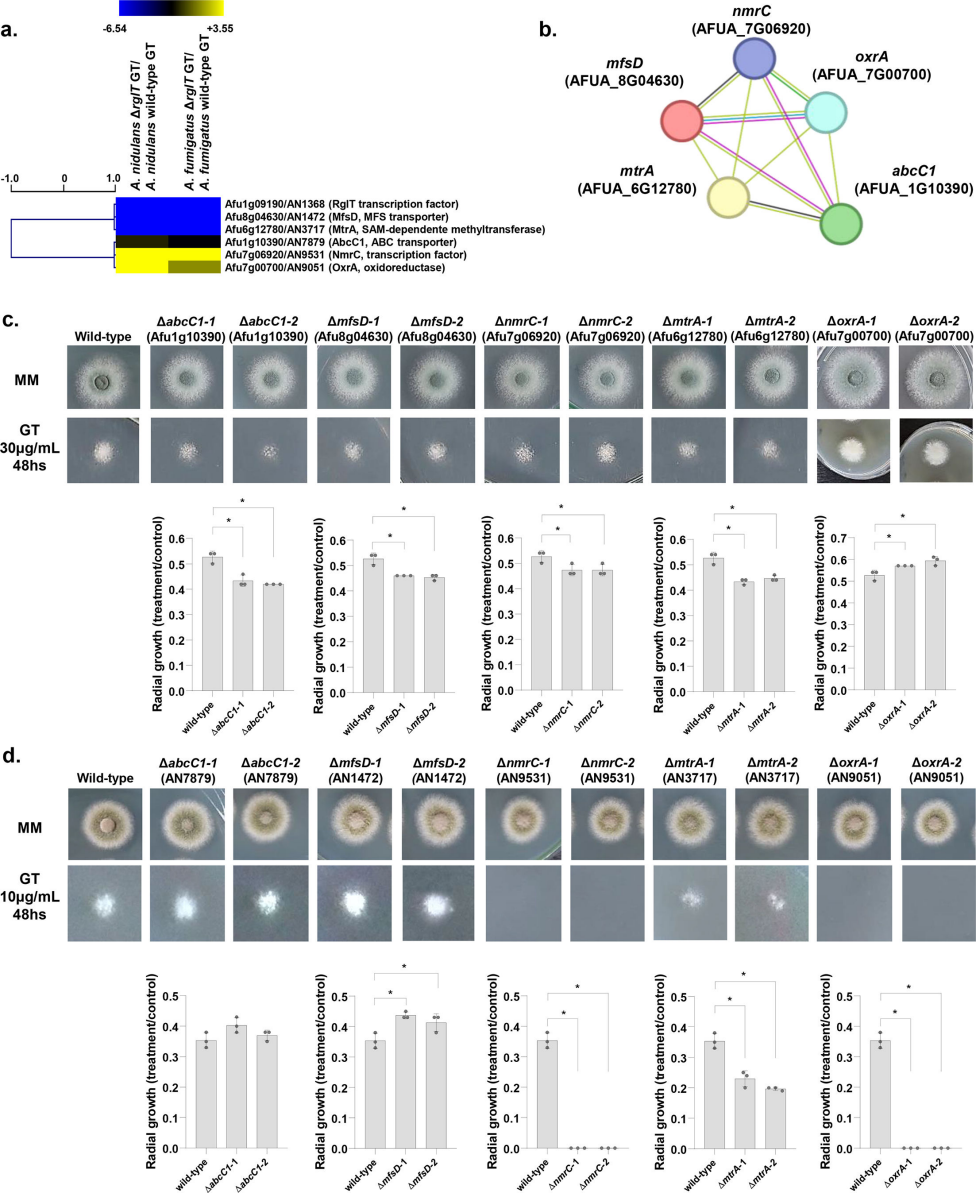
The identification of RglT-dependent *A. fumigatus* and *A. nidulans* homologs is important for GT self-protection. (**a**) Heatmap for the RNA-seq values of genes differentially expressed in the *A. fumigatus* and *A. nidulans* Δ*rglT* mutants when compared with the corresponding wild-type strains. Hierarchical clustering was performed in MeV (http://mev.tm4.org/), using Pearson correlation with complete linkage clustering. Differentially expressed genes (DEGs) were defined as those with a minimum of 2-fold change in gene expression (log2FC ≥ 1.0 and ≤−1.0; FDR of 0.05) when the Δ*rglT* mutants were compared with the wild-type strain under the equivalent conditions: *mfsD* (AFUA_8G04630 = −1.56; AN1472 = −5.44), *nmrC* (AFUA_7G06920 =+ 1,41; AN9531 =+ 3.55), *oxrA* (AFUA_7G00700 =+ 1.29; AN9051 =+ 1.57), *mtrA* (AFUA_6G12780 = −6.54; AN3717 = −5.51), and *abcC1* (AFUA_1G10390 =+ 1.10; AN7879 =+ 1.14). The differentially expressed genes are statistically significant (adjusted *P*-values < 0.005 for *A. fumigatus* and <0.05 for *A. nidulans*). (**b**) Functional protein association network, retrieved from STRING (https://string-db.org), shows that MtrA, OxrA, NmrC, AbcC1, and MfsD have functional associations (medium confidence score of 0.400). (**c and d**) Radial growth of *A. fumigatus* and *A. nidulans* wild type and mutants grown for 48 h at 37°C on MM in the presence and absence of GT. The results are the average of three repetitions ± standard deviation. Statistical analysis was performed using a one-tailed, paired *t*-test when compared with the control condition (*, *P* < 0.05).

Previously, aiming to determine a conserved transcriptional response to GT in both *A. fumigatus* and *A. nidulans*, we searched for homologous genes whose expression pattern was RglT-dependent in both fungal species in the presence of GT (6). In the comparison of differentially expressed genes (DEGs) from *A. fumigatus* Δ*rglT* mutant vs. wild-type with *A. nidulans* Δ*rglT* mutant vs wild-type, the following six homologs were differentially expressed in both species: *rglT* (AFUA_1G09190/AN1368), *mfsD* encoding a Major Facilitator Superfamily transporter (AFUA_8G04630/AN1472), *mtrA* encoding a methyltransferase (AFUA_6G12780/AN3717), *abcC1* encoding an ATP-binding cassette transporter (AFUA_1G10390/AN7879), *nmrC* encoding a transcription factor (AFUA_7G06920/AN9531), and *oxrA* encoding an oxidoreductase (AFUA_7G00700/AN9051) (Fig. 1a). Examination of the general *A. fumigatus* functional protein association network, retrieved from STRING (https://string-db.org), showed that these five *A. fumigatus* proteins have functional associations, suggesting they impact specific protein interaction networks (Fig. 1b). We constructed two independent mutants for each of these *A. fumigatus* and *A. nidulans* genes and determined their GT-susceptibility (Fig. 1c and d). We have used these two independent mutants to confirm that the observed phenotypic effects were not due to the occurrence of possible secondary mutations caused by the genetic transformation procedure. *A. fumigatus* Δ*abcC1*, Δ*mfsD*, Δ*nmrC*, and Δ*mtrA* mutants have about 20% radial growth reduction relative to the wild type under GT stress. By contrast, the Δ*oxrA* mutant exhibits ~13% greater radial growth than wild type under GT stress (Fig. 1c), indicating GT resistance. *A. nidulans* Δ*abcC1* is as GT-susceptible as the wild type (no growth difference), and the Δ*mfsD* mutant exhibits ~20% greater radial growth than wild type under GT treatment. In contrast, Δ*nmrC* and Δ*oxrA* show no growth in GT (high susceptibility), and Δ*mtrA* has ~40% less radial growth than the wild type on GT. (Fig. 1d). The *A. fumigatus* and *A. nidulans* Δ*mtrA* GT-susceptibilities were previously reported by (6).

Our results suggest that although there is a degree of conservation between the genes and their involvement in GT susceptibility, *A. nidulans* and *A. fumigatus* have distinct genetic programs to manage GT detoxification. This finding underscores the unique aspects of their responses and the need for further research to understand these specific genetic programs.

### GT and bmGT production

**Fig 2:**
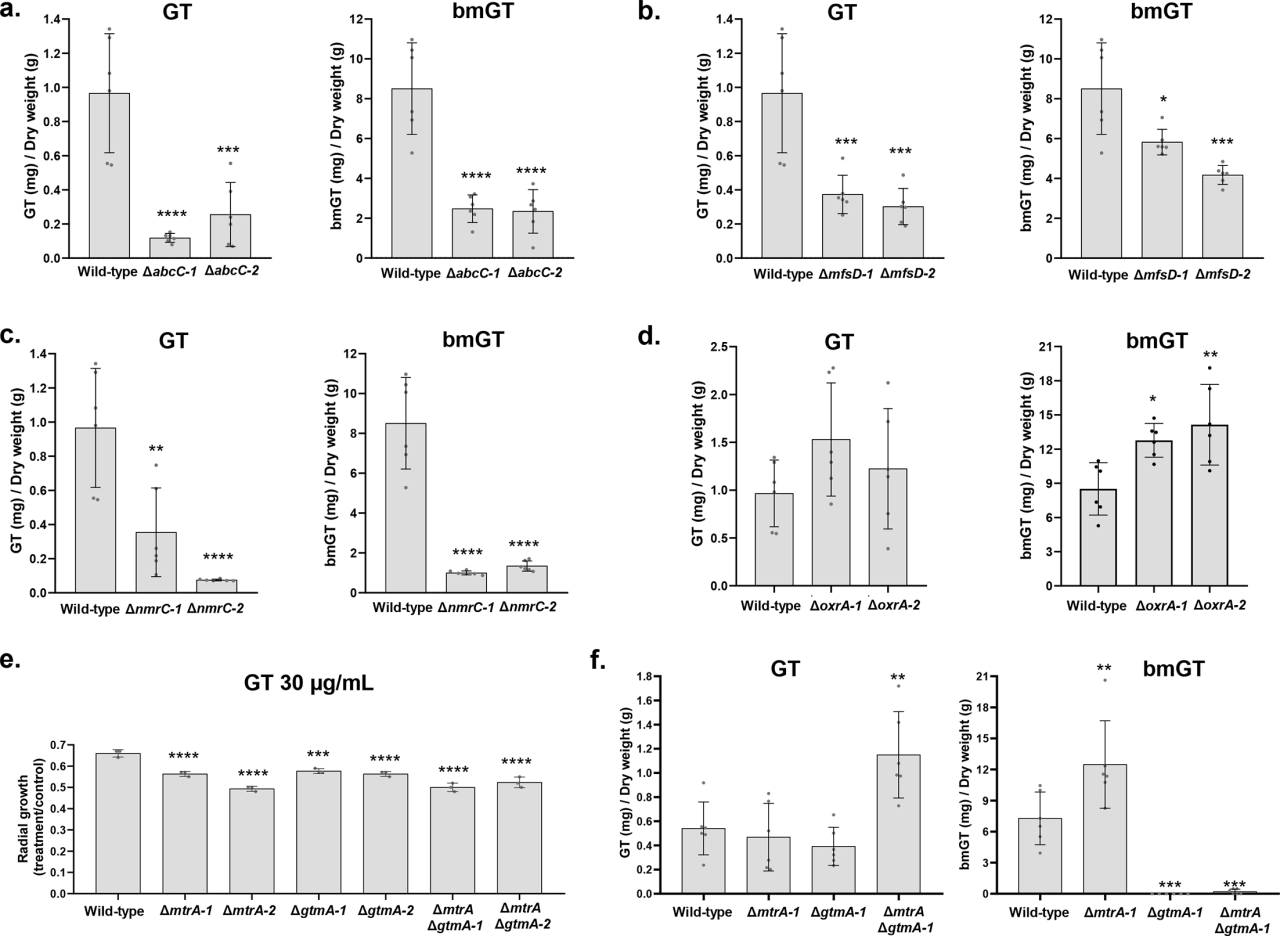
Production of GT and bmGT by *A. fumigatus* null mutants. (**a to d and f**) Relative abundance of GT and bmGT as measured by high-performance liquid chromatography coupled to mass spectrometry. Results are the average of three repetitions ± standard deviation. Statistical analysis was performed using one-way ANOVA followed by Tukey’s multiple comparisons test when comparing wild-type versus null mutants (*, *P* < 0.05; **, *P* < 0.01; ***, *P* < 0.001 and ****, *P* < 0.001). (**e**) Radial growth of *A. fumigatus* wild-type and mutants grown for 48 h at 37°C on MM in the presence and absence of GT. The results are the average of three repetitions ± standard deviation. Statistical analysis was performed using one-way ANOVA with Dunnett’s multiple comparisons test when comparing wild-type versus null mutants (***, *P* < 0.001 and ****, *P* < 0.0001).

Next, we determined GT and bmGT production in these *A. fumigatus* mutants. The Δ*abcC1*, Δ*mfsD*, and Δ*nmrC* have about 80%, 65%, and 80% less GT and 75%, 35%, and 90% less bmGT than the wild-type strain, respectively (Fig. 2a through c), whereas Δ*oxrA* produces GT at wild-type levels and about 20% more bmGT than wild type (Fig. 2d).

GtmA is a bis-thiomethyltransferase that can convert dtGT into bisdethiobis(methylthio)-gliotoxin (bmGT) and attenuate GT production postbiosynthetically (9). We constructed a double mutant Δ*mtrA* Δ*gtmA,* aiming to determine if there are any genetic interactions between *mtrA* and *gtmA* (Fig. 2e). The single and double mutants have a comparable GT-susceptibility, that is, about 20% more GT-susceptible than the wild-type strain (Fig. 2e). The Δ*mtrA* and Δ*gtmA* mutants have comparable GT production to the wild-type strain, but Δ*mtrA* Δ*gtmA* has about 2-fold more GT production than the wild-type (Fig. 2f). The Δ*mtrA* produces nearly 2-fold more bmGT than the wild-type strain, but Δ*gtmA* and the double mutant Δ*mtrA* Δ*gtmA* have no bmGT production (Fig. 2f). Considering that (i) there is an increased accumulation of GT in the double mutant and no differences between the wild-type and Δ*mtrA* in GT production, and (ii) there is an increased accumulation of bmGT in the Δ*mtrA* mutant and no bmGT in the double mutant, these results suggest that MtrA and GtmA are acting independently on the regulation of GT and bmGT production.

### Phenotypic characterization of Δ*mtrA* and Δ*nmrC* mutants

**Fig 3:**
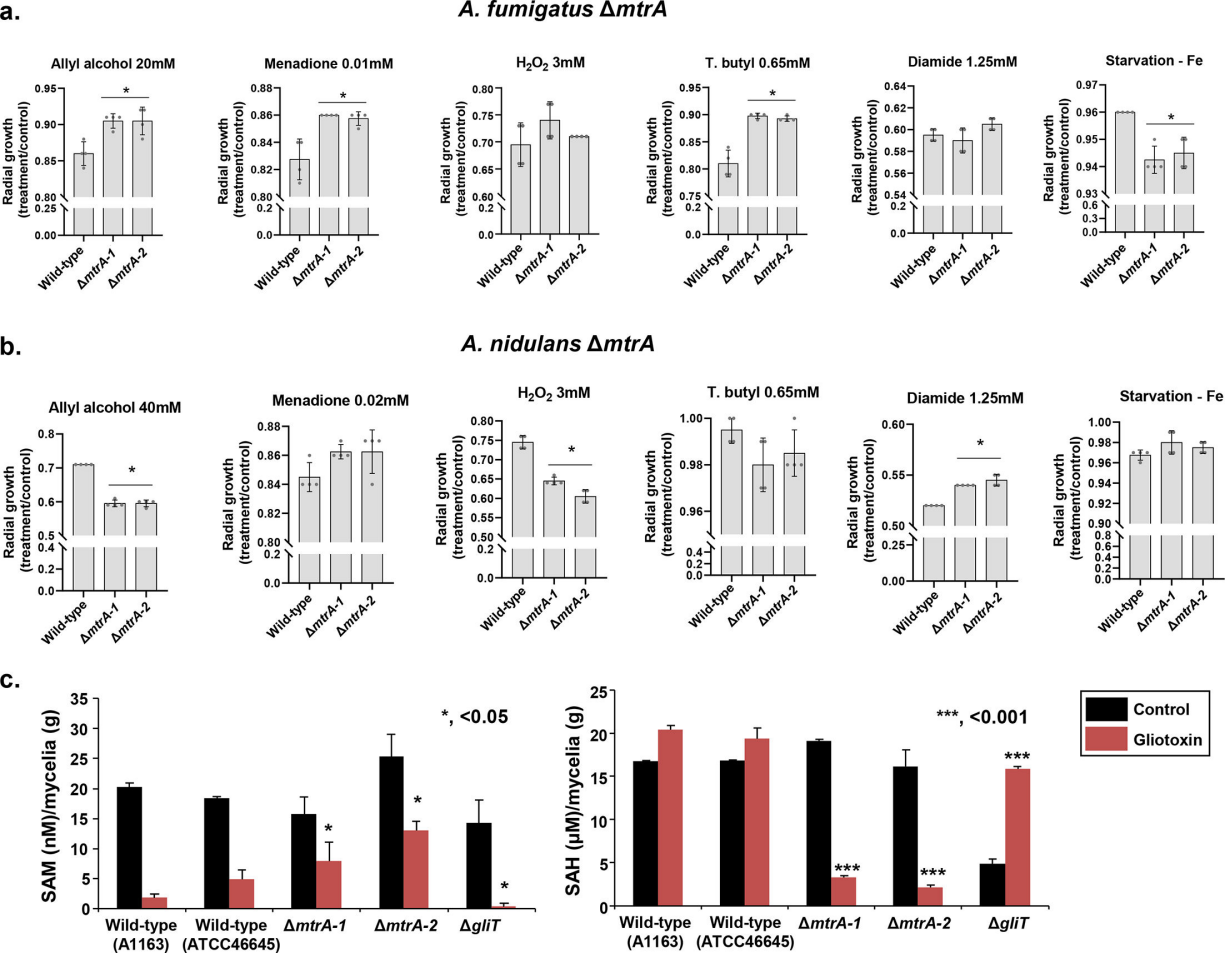
Phenotypic characterization of Δ*mtrA* mutants. (**a and b**) Radial growth of *A. fumigatus* and *A. nidulans* wild-type and Δ*mtrA* mutants grown for 5 days at 37°C on MM in the absence and presence of different stressing agents. Results are the average of three repetitions ± standard deviation. Statistical analysis was performed using one-way ANOVA with Dunnett’s multiple comparisons test when comparing wild-type versus null mutants (*, *P* < 0.01). (**c**) SAM and SAH levels were measured in the wild-type, Δ*mtrA*, and Δ*gliT* mutants. The strains were grown in liquid MM for 24 h at 37°C and exposed to GT 5 µg/mL for 3 h. Results are the average of three repetitions ± standard deviation. Statistical analysis was performed using a one-tailed, paired *t*-test when compared with the control condition (*, *P* < 0.05; ***, *P* < 0.001).

**Fig 4:**
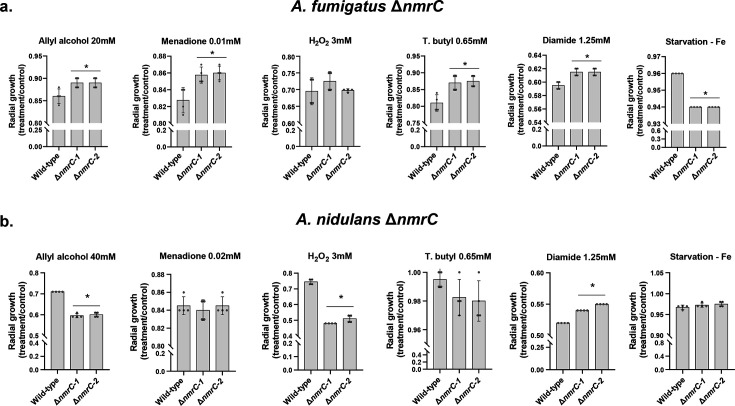
Phenotypic characterization of Δ*nmrC* mutants. (**a and b**) Radial growth of *A. fumigatus* and *A. nidulans* wild-type and Δ*nmrC* mutants grown for 5 days at 37°C on MM in the absence and presence of different stressing agents. Results are the average of three repetitions ± standard deviation. Statistical analysis was performed using one-way ANOVA with Dunnett’s multiple comparisons test when comparing wild-type versus null mutants (*, *P* < 0.01).

We extensively phenotyped all five *A. fumigatus* and *A. nidulans* null mutants to susceptibility to oxidative stress (allyl alcohol, menadione, H_2_O_2_, and tert-butyl hydroperoxide) iron starvation (bathophenanthrolinedisulfonate, BPS) and excess, zinc starvation (phenanthroline) and excess, diamide (a glutathione scavenger), and growth on different single sources of sulfur (inorganic, methionine, and cysteine). We did not observe any differences in these phenotypes in *A. fumigatus* and *A. nidulans* Δ*mfsD* and Δ*oxrA* when compared with the corresponding wild-type strains. However, Δ*mtrA*, Δ*abcC1*, and Δ*nmrC* showed some distinct phenotypic differences compared with the wild-type strain (Fig. 3 and 4; see Fig. S1 at https://doi.org/10.6084/m9.figshare.29825336.v3). The *A. fumigatus* and *A. nidulans* Δ*abcC1* mutants are more sensitive and resistant, respectively, to the glutathione (GSH) scavenger diamide (see Fig. S1a at https://doi.org/10.6084/m9.figshare.29825336.v3). Accordingly, there is an increase in the total GSH when *A. fumigatus* wild type is exposed to 5 µg/mL of GT for different periods, and there is a decrease in the GSH content in Δ*abcC1* mutants when exposed to GT (see Fig. S1b at https://doi.org/10.6084/m9.figshare.29825336.v3). Interestingly, the *A. fumigatus* Δ*rglT* also has decreased GSH concentrations when exposed to GT (see Fig. S1c at https://doi.org/10.6084/m9.figshare.29825336.v3). There are no differences between the wild-type strain, Δ*mfsD*, and Δ*abcC1* regarding the minimal inhibitory concentration (MIC) to azoles or the minimal effective concentration (MEC) to caspofungin, suggesting that these transporters are not multidrug transporters.

The *A. fumigatus* Δ*mtrA* mutants are more resistant to allyl alcohol, menadione, and tert-butyl hydroperoxide and more sensitive to iron starvation (Fig. 3a). In contrast, the *A. nidulans* Δ*mtrA* mutants are more sensitive to allyl alcohol, H_2_O_2_, and more resistant to diamide (Fig. 3b). In contrast to what was previously observed for other *A. fumigatus* clinical isolates (18), exposure to exogenous GT has a significant impact on the levels of S-adenosylmethionine (SAM) and S-adenosylhomocysteine (SAH) of the *A. fumigatus* A1163 and ATCC46645 clinical isolates (Fig. 3c). As expected, Δ*gliT* (whose parent strain is ATCC46645) has very low amounts of SAM and increased amounts of SAH upon GT exposure (Fig. 3c). We also determined the effect of *mtrA* (whose parent strain is A1163) null mutations on SAM and SAH levels in *A. fumigatus*. In the wild-type strain, GT exposure causes SAM levels to decrease and SAH levels to increase (relative to unexposed control) (Fig. 3c). In Δ*mtrA* mutants under GT, SAM levels remain higher (and SAH lower) than those in the GT-treated wild type. These results suggest that the lack of *mtrA* affects SAM and SAH homeostasis upon GT exposure.

The *A. fumigatus* Δ*nmrC* mutants are more resistant to allyl alcohol, menadione, tert-butyl hydroperoxide, and diamide and more sensitive to iron starvation (Fig. 4a). In contrast, the *A. nidulans* Δ*nmrC* mutants are more sensitive to allyl alcohol and H_2_O_2_ and more resistant to diamide (Fig. 4b).

### MtrA and NmrC exhibit both shared and distinct transcriptional signatures

**Fig 5:**
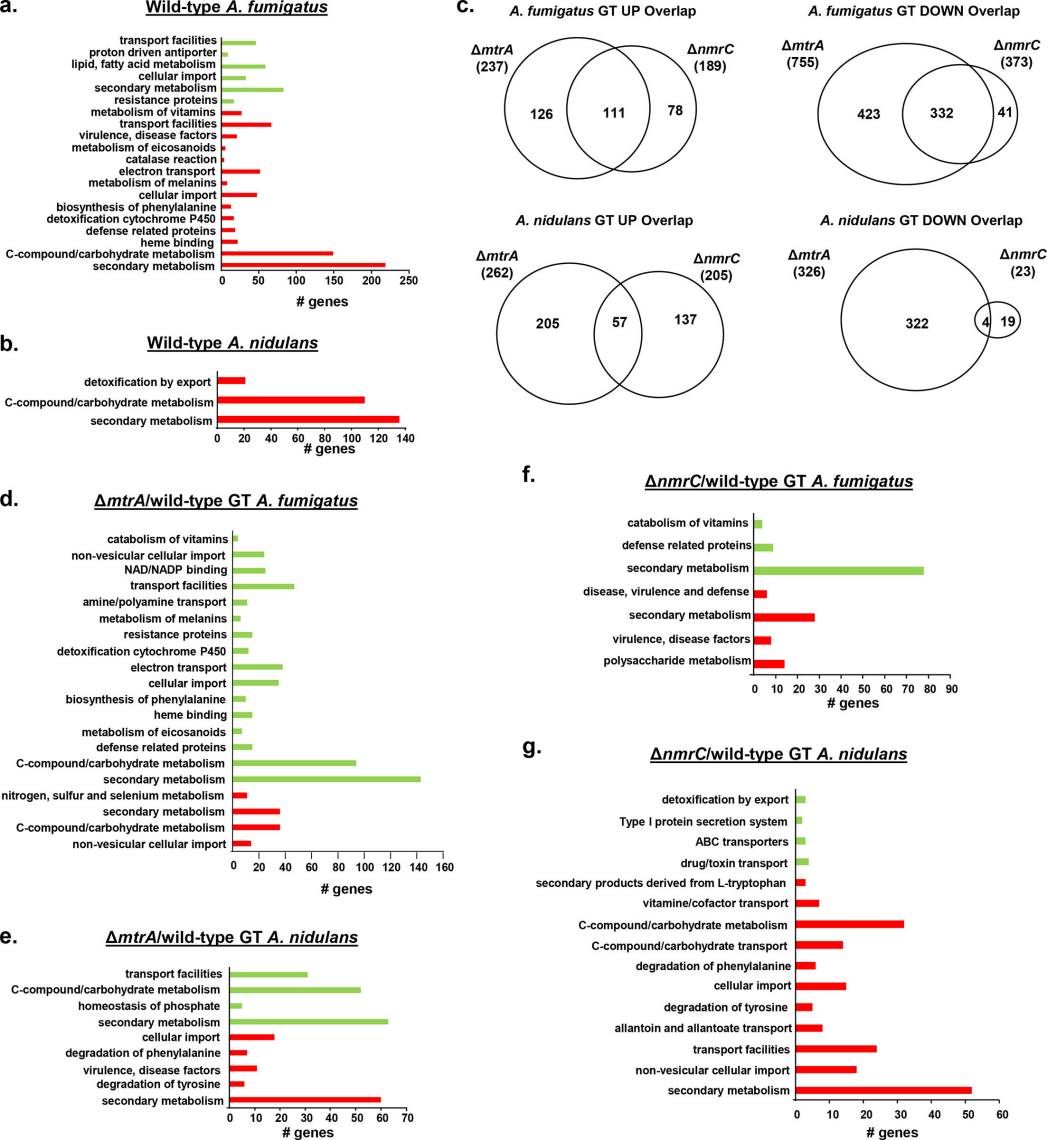
MtrA and NmrC have common transcriptional targets. (**a**) Venn diagram for genes differentially expressed (upregulated and downregulated) in the *A. fumigatus* and *A. nidulans* wild-type, Δ*mtrA*, and Δ*nmrC* when exposed to GT 5 µg/ml for 3 h. (**b to g**) A summary of the FunCat terms over-represented up (red color) or down (green color) regulated (adjusted *P*-value < 0.05) in log2FC *A. fumigatus* or *A. nidulans* wild-type or mutants exposed to GT 5 µg/mL for 3 h.

The *A. fumigatus* and *A. nidulans* wild-type, Δ*mtrA*, and Δ*nmrC* transcriptomes were assessed post-exposure to 5 µg/mL of GT for 3 h, as previously described (6), aiming to identify MtrA-dependent and NmrC-dependent gene expression. Differentially expressed genes (DEGs) were defined as those with a minimum of 2-fold change in gene expression (log2FC ≥ 1.0 and ≤−1.0; FDR of 0.05) when compared with the wild-type strain under the equivalent conditions. The *A. fumigatus* wild-type has 1,180 and 663 genes up- and down-regulated, respectively, and *A. nidulans* wild-type has 746 and 399 genes up- and down-regulated, respectively, when these strains were exposed to GT (see Table S1 at https://doi.org/10.6084/m9.figshare.29825336.v3). There are 75 and 55 homologs of both species up- and down-regulated upon exposure to GT (see Table S1 at https://doi.org/10.6084/m9.figshare.29825336.v3). Funcat enrichment analysis (https://fungifun3.hki-jena.de/) for the *A. fumigatus* wild-type showed upregulation of secondary metabolism, C-compound and carbohydrate metabolism, electron transport, and transport facilities and downregulation of lipid, fatty acid, and isoprenoid metabolism (Fig. 5a). Categorization for the *A. nidulans* wild-type showed upregulation of detoxification by export, secondary metabolism, and C-compound and carbohydrate metabolism; there is no enrichment for *A. nidulans* downregulated genes (Fig. 5b). Previously, we have observed that the deletion of the *A. fumigatus* homolog of the transcription factor Yap1, a master regulator of oxidative stress, is more sensitive to GT (6). However, we have not observed any transcriptional modulation of *A. fumigatus* and *A. nidulans* Yap1 homologs in the RNA-seq analysis (see Table S1 at https://doi.org/10.6084/m9.figshare.29825336.v3).

The *A. fumigatus* Δ*mtrA* has 237 and 755 up- and down-regulated genes, respectively, and *A. nidulans* Δ*mtrA* has 262 and 326 up- and down-regulated genes, respectively, when these strains were exposed to GT (Fig. 5c; see Table S1 at https://doi.org/10.6084/m9.figshare.29825336.v3). The *A. fumigatus* Δ*nmrC* has 189 and 373 up- and down-regulated genes, respectively, and *A. nidulans* Δ*nmrC* has 149 and 417 up- and down-regulated genes, respectively, when these strains were exposed to GT (Fig. 5c; see Table S1 at https://doi.org/10.6084/m9.figshare.29825336.v3). Venn analyses of the genes differentially expressed in *A. fumigatus* Δ*mtrA* and Δ*nmrC* versus the wild-type strain under GT exposure revealed a complex pattern of shared and mutant-specific transcriptional responses to GT stress (Fig. 5c). The *A. fumigatus* Δ*mtrA* and Δ*nmrC* mutants shared 111 and 332 up- or down-regulated genes, respectively, whereas *A. nidulans* shared 57 and 4 up- or down-regulated genes, respectively (Fig. 5c). Mutant-specific transcriptional responses were more prevalent, with *A. fumigatus* exhibiting 126 and 423 uniquely up- and down-regulated genes, respectively, for Δ*mtrA*, and 78 and 41 uniquely up- and down-regulated genes, respectively, for Δ*nmrC* (Fig. 5c). *A. nidulans* showed 205 and 322 uniquely up- and down-regulated genes, respectively, for Δ*mtrA*, and 137 and 19 uniquely up- and down-regulated genes, respectively, for Δ*nmrC* (Fig. 1c). FunCat enrichment analyses for *A. fumigatus* Δ*mtrA* mutant showed up- and down-regulated secondary metabolism and C-compound and carbohydrate metabolism, and down-regulated electron transport (Fig. 5d). *A. nidulans* Δ*mtrA* has enrichment for the upregulation of secondary metabolism, cellular import, and degradation of tyrosine and phenylalanine and downregulation of secondary metabolism, transport facilities, and C-compound and carbohydrate metabolism (Fig. 5e). FunCat enrichment analyses for *A. fumigatus* Δ*nmrC* mutant showed up- and down-regulated secondary metabolisms, whereas *A. nidulans* Δ*nmrC* has enrichment for the upregulation of secondary metabolism, C-compound and carbohydrate metabolism, transport facilities, and degradation of tyrosine and phenylalanine and downregulation of ABC transporters (Fig. 5f and g). The log2 differential expression of *gliT* and *gtmA* when the wild-type is exposed to GT is +4.19 and +4.49 (see Table S1 at https://doi.org/10.6084/m9.figshare.29825336.v3). However, there is no differential expression in the *gliT* and *gtmA* between the wild-type, Δ*mtrA*, and Δ*nmrC* (see Table S1 at https://doi.org/10.6084/m9.figshare.29825336.v3). The *gliT* and *gtmA* expressions in the Δ*mfsD*, Δ*oxrA*, and Δ*abcC1* need to be determined.

These results indicate that GT is affecting both species in terms of secondary metabolism, C-compound, and carbohydrate metabolism. Several *A. fumigatus* and *A. nidulans* homologs are concomitantly differentially expressed upon GT exposure. Moreover, Δ*mtrA* and Δ*nmrC* mutants share a subset of target genes and functional categories, suggesting that MtrA and NmrC function in overlapping or related pathways downstream of RglT.

### *A. fumigatus* and *A. nidulans* MtrA and NmrC proteins show conserved functional associations with mitochondrial proteins

**Fig 6:**
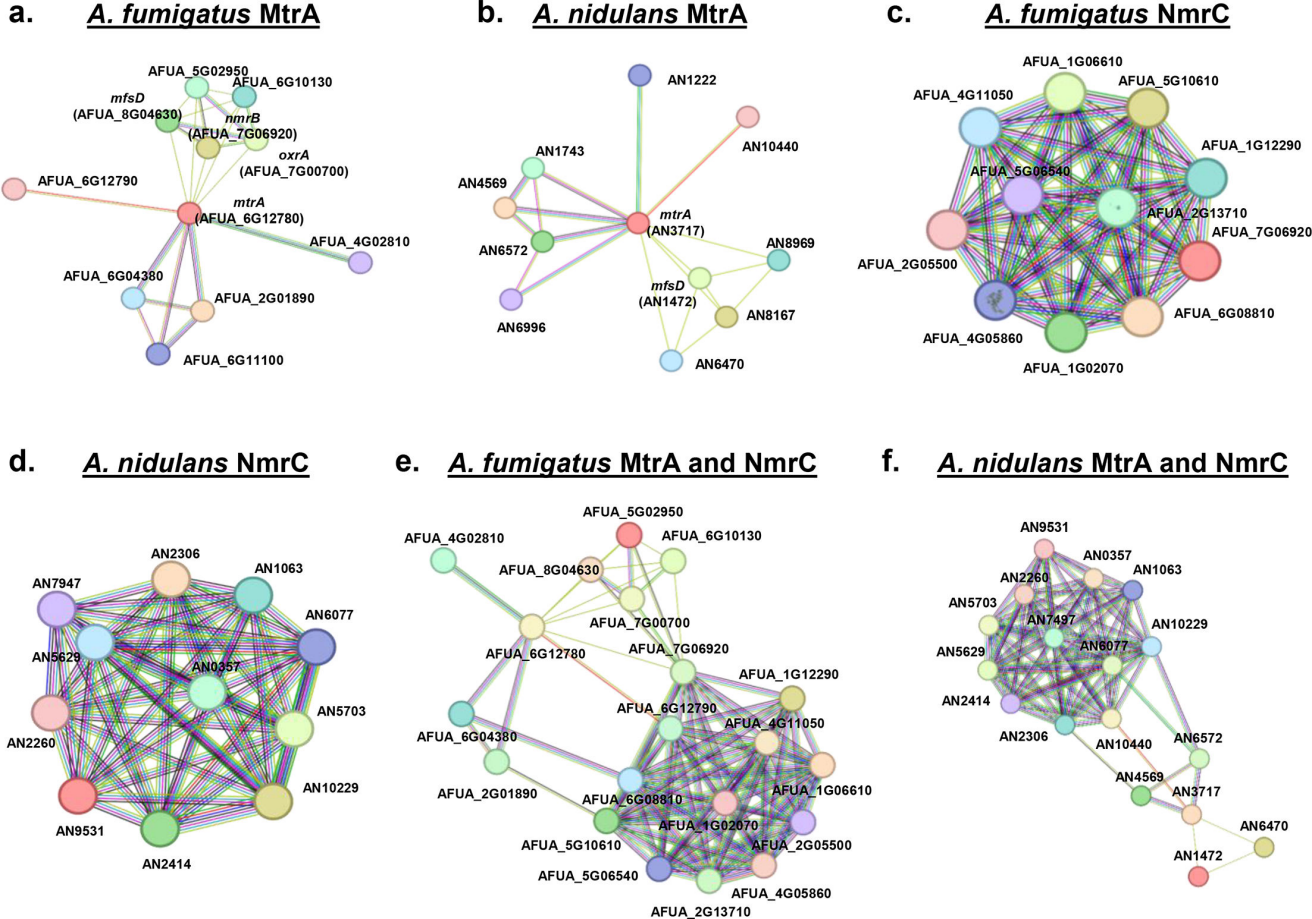
*A. fumigatus* and *A. nidulans* MtrA and NmrC protein-protein interactions. (**a**) *A. fumigatus* MtrA. (**b**) *A. nidulans* MtrA. (**c**) *A. fumigatus* NmrC. (**d**) *A. nidulans* NmrC. (**e**) *A. fumigatus* MtrA and NmrC. (**f**) *A. nidulans* MtrA and NmrC. The analysis was performed by using STRING (https://string-db.org/) (medium confidence scores of 0.400).

We used STRING once more to assess the possible protein interaction networks by looking at proteins that interact either with *A. fumigatus* or *A. nidulans* MtrA/NmrC (Fig. 6; Table 1). *A. fumigatus* MtrA interacts with 10 proteins, including OxrA and NmrC, whereas *A. nidulans* MtrA also interacts with 10 proteins, but only six of those have homologs among the 10 *A*. *fumigatus* proteins (Fig. 6a and b; Table 1). *A. fumigatus* and *A. nidulans* NmrC interact with 10 proteins, 9 of which are homologs (Fig. 6c and d; Table 1). Of these 20 proteins (10 from MtrA and 10 from NmrC), 15 are related to mitochondrial function, and 12 are directly related to ubiquinone biosynthesis. All these *A. fumigatus* MtrA and NmrC and *A. nidulans* MtrA and NmrA proteins show protein-protein interactions (Fig. 6e and f; Table 1). Notice that the MfsD and AbcC1 were shown as interacting proteins when only the five proteins were used as queries in the STRING (Fig. 1b). However, when either MtrA or NmrC is independently used as a query, the two proteins do not show as interacting proteins. Our results suggest that *A. fumigatus* and *A. nidulans* MtrA and NmrC associate functionally with mitochondrial proteins, particularly those in ubiquinone (coenzyme Q) biosynthesis.

**TABLE 1:** *A. fumigatus* and *A. nidulans* MtrA and NmrC protein-protein interactions

*A.fumigatus*	*A. nidulans*	Function
AFUA_6 G12780	AN3717	MtrA methyl transferase
AFUA_4G02810	Not observed	Oxidoreductase
AFUA_6G11100	Not observed	Methyl transferase (Coq5)
AFUA_2G01890	AN4569	5-demethoxyubiquinone hydroxylase (Coq7)
AFUA_6G04380	AN6572	ATPase required for ubiquinone biosynthesis
AFUA_6G12790	AN10440	NADH dehydrogenase (ubiquinone) 1 alpha subcomplex 9
AFUA_8G04630	AN1472	C4-dicarboxylate transporter
AFUA_7G06920	Not observed	Transcription factor NmrC
AFUA_7G00700	Not observed	Aldo keto reductase OxrA
AFUA_5G02950	AN1472	DUF1996 domain-containing protein
AFUA_6G10130	AN6470	N,O-diacetyl muramidase
AFUA_7 G06920	AN9531	Transcription factor NmrC
AFUA_1G06610	AN5703	NADH-ubiquinone oxidoreductase
AFUA_5G10610	AN2306	Cytochrome b-c1 complex subunit Rieske, mitochondrial
AFUA_1G12290	AN1063	NADH-ubiquinone oxidoreductase
AFUA_5G06540	AN2260	NADH-ubiquinone oxidoreductase subunit B17.2
AFUA_6G08810	AN10229	NADH dehydrogenase (ubiquinone)
AFUA_1G02070	AN0357	Cytochrome C1
AFUA_4G05860	Not observed	NADH-ubiquinone oxidoreductase subunit B1
AFUA_2G05500	AN7497	NADH-ubiquinone oxidoreductase 18 kDa subunit
AFUA_4G11050	AN5629	NADH-ubiquinone oxidoreductase subunit F
AFUA_2G13710	AN2414	NADH-ubiquinone oxidoreductase 49 kDa
Not observed	AN6077	NADH dehydrogenase (ubiquinone)

We also examined the genomic synteny surrounding *mtrA* and *nmrC* in both *A. fumigatus* and *A. nidulans*. This analysis revealed that *mtrA* is flanked by a gene encoding a putative NADH-ubiquinone oxidoreductase 39 kDa subunit in both species—AFUA_6G12790 in *A. fumigatus* and AN10440 in *A. nidulans* (see Fig. S2 at https://doi.org/10.6084/m9.figshare.29825336.v3). Notably, this NADH-ubiquinone oxidoreductase was also identified in the STRING protein interaction networks for both methyltransferases (Table 1). In contrast, the genomic region surrounding *nmrC* exhibited less conserved synteny between the two species (see Fig. S2 at https://doi.org/10.6084/m9.figshare.29825336.v3).

To test this possible interaction between MtrA, NmrC, and mitochondrial proteins involved in ubiquinone (coenzyme Q) biosynthesis, we concentrated our analysis on *A. fumigatus*. Recently, we identified 468 nuclear-encoded mitochondrial (NEM) ortholog genes in *A. fumigatus* (4). We compared all the differentially expressed genes in our RNAseq when *A. fumigatus* wild-type, Δ*mtrA*, and Δ*nmrC* mutants were exposed to GT 5 µg/mL for 3 h with these 468 NEM genes (Table 2; see Table S2 at https://doi.org/10.6084/m9.figshare.29825336.v3). There are 109 of these 468 NEM genes (22%) that are differentially expressed when the wild-type strain is exposed to GT (Table 2; see Table S2 at https://doi.org/10.6084/m9.figshare.29825336.v3). In contrast, there were 39 (8 %) and 14 (3 %) when the Δ*mtrA* and Δ*nmrC* were exposed to GT, respectively (Table 2; see Table S2 at https://doi.org/10.6084/m9.figshare.29825336.v3). These results suggest that GT can modulate the expression of a substantial number of NEM genes, and both MtrA and NmrC play a role in this process.  

**TABLE 2:** Number of nuclear-encoded mitochondrial genes modulated by GT

	Total DE	Mitochondrial DE^*a*^	Mitochondrial UP	Mitochondrial DOWN
Wild-type GT/wild-type	3338	105 (22 %)	48	57
Δ*mtrA* GT/wild-type GT	2014	39 (8 %)	22	17
Δ*nmrC* GT/wild-type GT	1120	14 (3 %)	9	5

^*a*^ DE = Differentially expressed genes. There are 468 nuclear-encoded mitochondrial genes.

**Fig 7:**
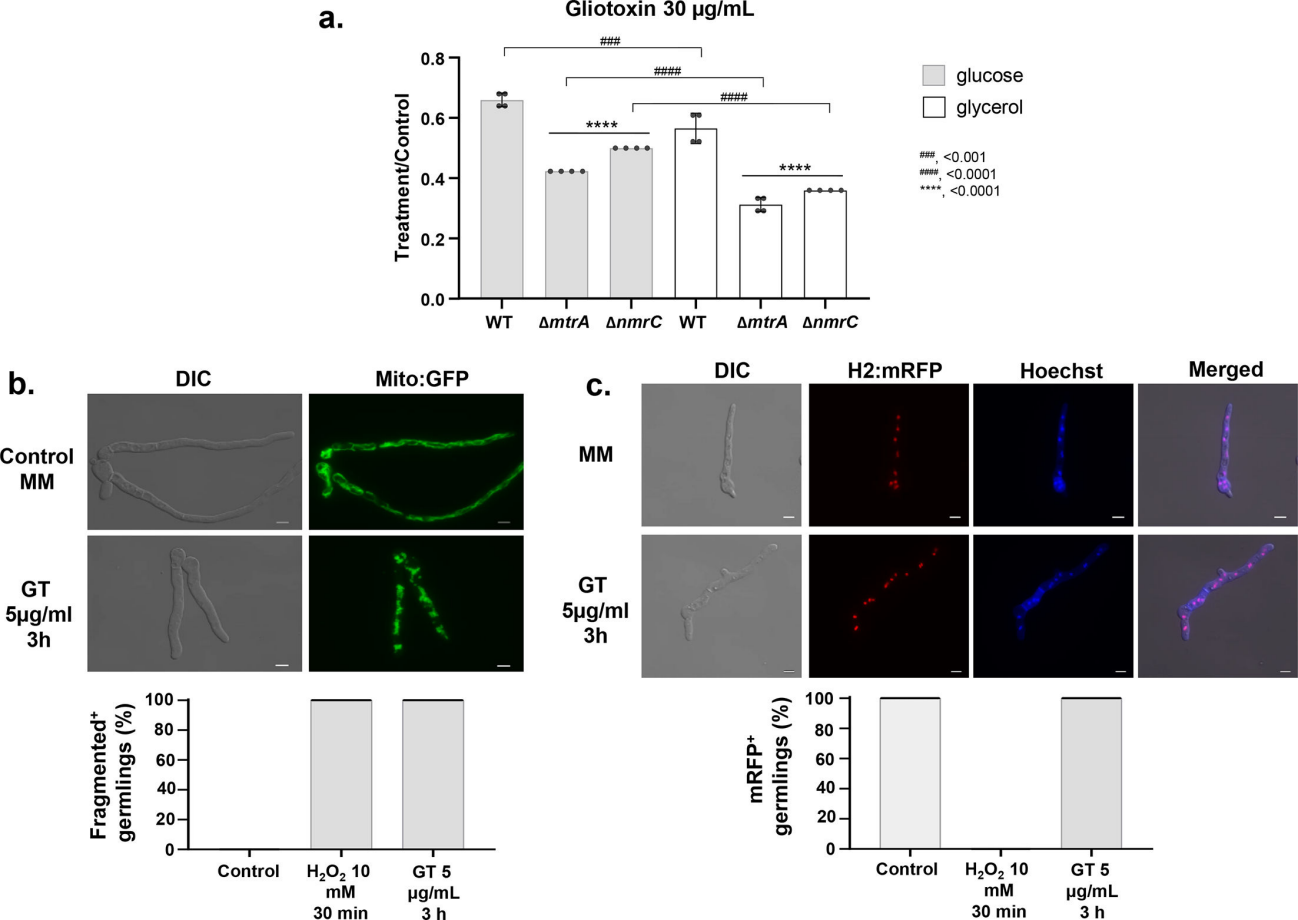
GT affects the mitochondrial function and induces cell death. (**a**) *A. fumigatus* was grown for 48 h at 37°C in MM + glucose or MM + glycerol supplemented or not with GT 30 µg/mL. Results are the average of three repetitions ± standard deviation. Statistical analysis was performed using one-way ANOVA followed by Tukey’s multiple comparisons test. Significant differences between the wild-type and mutant strains are indicated by ****, *P* < 0.0001, whereas comparisons between glucose and glycerol conditions for the same strain are indicated by ####, *P* < 0.0001 and ###, *P* < 0.001. (**b**) An *A. fumigatus* strain with mitochondria constitutively expressing GFP was grown for 16 h at 37°C and exposed or not to H_2_O_2_ 10 mM for 30 min or GT 5 µg/mL for 3 h. The results show the percentage of germlings with fragmented mitochondria and are expressed as the average of three repetitions with a total of 100 germlings ± standard deviation. A representative image for control (MM) and GT treatment is shown, scale bar = 5 µm. (**c**) An *A. fumigatus* strain containing the histone h2A::RFP was grown for 16 h at 37°C and exposed or not to H_2_O_2_ 10 mM for 30 min or GT 5 µg/mL for 3 h. The results show the percentage of germlings with Hoechst staining and RFP and are expressed as the average of three repetitions with a total of 100 germlings ± standard deviation. A representative image for control (MM) and GT treatment is shown, scale bar = 5 µm.

Next, we have grown *A. fumigatus* wild-type, Δ*mtrA*, and Δ*nmrC* and determined the GT susceptibility on MM + glucose (a fermentable carbon source) or MM + glycerol (a non-fermentable carbon source) as single carbon sources (Fig. 7a). The wild-type, Δ*mtrA*, and Δ*nmrC* strains are more sensitive to GT (radial growth inhibition) when grown on MM + glycerol than on MM + glucose (Fig. 7a), supporting the idea that GT compromises mitochondrial function (since a non-fermentable carbon source requiring respiration led to greater sensitivity). Fungal mitochondria without any stress are seen enriched as tubular and dynamic networks, but in the presence of antifungals or other stresses, the mitochondria begin to fragment, which is an indicator of cellular death (19, 20) (see a representative image in Fig. 7b). No germlings showed fragmented mitochondria in untreated controls, whereas nearly 100% showed fragmentation after GT treatment (Fig. 7b). The activation of fungal metacaspases, for example, during oxidative stress, induces markers of apoptosis-like cell death such as nuclear condensation, disorganization of the histone complex, and DNA double-strand breaks, which coincide with the loss of fungal cell viability (21). A fluorescent histone 2A construct (h2A::mRFP) has been used as a marker of cell death in *A. fumigatus* (22). To check if the GT induced apoptosis-like cell death, germlings of the *A. fumigatus* h2A::mRFP strain were exposed to H_2_O_2_ (10 mM) as a positive control for 1 h or to GT 5 µg/mL for 3 h. The nuclei were stained with Hoechst. The H_2_O_2_ positive control showed complete loss of red fluorescence, indicative of a disorganized histone complex (a representative image is shown in Fig. 7c). However, no differences in mRFP fluorescence were observed in the germlings exposed to GT (Fig. 7c). Overall, these results suggest that GT causes cell death of *A. fumigatus* by damaging the mitochondria but does not induce apoptosis-like cell death.

To further investigate possible interactions between GT, wild-type, Δ*mtrA*, and Δ*nmrC* mutants, we determined the minimal inhibitory concentration (MICs) of mitochondrial inhibitors, such as antimycin A, which blocks electron transport between coenzyme Q and cytochrome c, a classical Complex III inhibitor, https://pubchem.ncbi.nlm.nih.gov/compound/antimycin-A#section=Mechanism-of-Action; salicylhydroxamic acid, SHAM, an alternative oxidase inhibitor (23); sodium azide, which blocks the oxygen-binding pocket in the active center of cytochrome oxidase, https://pubchem.ncbi.nlm.nih.gov/compound/33557#section=Use-Classification; or sodium nitroprusside, a nitric oxide donor that affects *S. cerevisiae* mitochondrial function (24), alone or in combination with GT for the *A. fumigatus* wild-type, Δ*mrtA*, and Δ*nmrC* (Table 3). As previously shown, the GT MICs for wild type = 70 µg/mL and for both Δ*mtrA* and Δ*nmrC* = 35 µg/mL (Table 3). There is an interaction between GT and antimycin A in the wild-type and mutants. Antimycin A MIC (alone) is ~0.312 mM for the wild type and ~0.156 mM for the mutants. In combination with GT, a much lower antimycin A concentration (~0.0024 mM) inhibits mutant growth (Table 3), versus ~0.156 mM required for wild type (Table 3). The MIC for sodium azide for all the strains was 0.62 mM, and there is an interaction between GT and sodium azide for all the strains (8.75 µg/mL GT + 0.039 mM sodium azide; Table 3). The MICs for SHAM are 62.5, 3.9, and 7.81 for the wild-type, Δ*mtrA,* and Δ*nmrC*, respectively (Table 3). Drug interaction is also observed for SHAM, where combinations of 35 µg/mL GT +0.24 mM SHAM can inhibit the wild-type growth, whereas Δ*mtrA* and Δ*nmrC* can be inhibited by 4.4 µg/mL GT +0.98 mM and 8.75 µg/mL GT +0.98 mM SHAM, respectively (Table 3). The MICs for sodium nitroprusside for the wild-type and the mutants are 125 mM, and the concentrations to inhibit the wild-type, Δ*mtrA*, and Δ*nmrC* are 35.0 µg/mL +0.97 mM and 17.5 µg/mL + 3.9 mM (Table 3).

**TABLE 3:** *A. fumigatus* MIC for GT, mitochondrial inhibitors, and a nitric oxide donor^*a*^

	GT (µg/mL)	Antimycin A (mM)	GT (µg/mL) + Antimycin A (mM)	Interaction between GT and other drugs
Wild-type	70	0.312	35.0 + 0.0024	Yes
Δ*mtrA*-1	35	0.156	35.0 + 0.156	Yes
Δ*mtrA-2*	35	0.156	35.0 + 0.156	Yes
Δ*nmrC*-1	35	0.156	35.0 + 0.156	Yes
Δ*nmrC-2*	35	0.156	35.0 + 0.156	Yes
Δ*aoxA*	70	0.078	35.0 + 0.0012	Yes

	GT	Sodium azide (mM)	GT (µg/mL) + Sodium azide (mM)	
Wild-type	70	0.62	8.75 + 0.039	Yes
Δ*mtrA*-1	35	0.62	8.75 + 0.039	Yes
Δ*mtrA-2*	35	0.62	8.75 + 0.039	Yes
Δ*nmrC*-1	35	0.62	8.75 + 0.039	Yes
Δ*nmrC-2*	35	0.62	8.75 + 0.039	Yes
Δ*aoxA*	70	0.62	8.75 + 0.039	Yes

	GT	SHAM (mM)	GT (µg/mL) + SHAM (mM)	
Wild-type	70	62.5	35.0 + 0.24	Yes
Δ*mtrA*-1	35	3.9	4.4 + 0.98	Yes
Δ*mtrA-2*	35	3.9	4.4 + 0.98	Yes
Δ*nmrC*-1	35	7.81	8.75 + 0.98	Yes
Δ*nmrC-2*	35	7.81	8.75 + 0.98	Yes
Δ*aoxA*	70	62.5	35.0 + 0.24	Yes

	GT	Sodium nitroprusside (mM)	GT (µg/mL) + Sodium nitroprusside (mM)	
Wild-type	70	125	35.0 + 0.97	Yes
Δ*mtrA*-1	35	125	17.5 + 0.97	Yes
Δ*mtrA-2*	35	125	17.5 + 0.97	Yes
Δ*nmrC*-1	35	125	17.5 + 3.9	Yes
Δ*nmrC-2*	35	125	17.5 + 3.9	Yes
Δ*aoxA*	70	125	35.0 + 31.25	Yes

^*a*^ Three repetitions were used for each treatment.

Alternative oxidase (AOX) is a mitochondrial respiratory enzyme found in many fungi that provides an alternative pathway for electron transport, bypassing complexes III and IV of the classical electron transport chain. This cyanide-insensitive pathway helps maintain redox balance and ATP production under stress conditions, such as oxidative stress, antifungal exposure, or respiratory chain inhibition. AOX has been implicated in fungal virulence and drug tolerance, making it a potential target for antifungal therapy (25, 26). We also observed that the *A. fumigatus* Δ*aoxA* (*aoxA* encodes the alternative oxidase) was more sensitive to antimycin A and had a stronger inhibitory interaction between GT and antimycin A than the wild-type (Table 3).

In summary, compromising mitochondrial function (via electron transport inhibitors or an NO donor) greatly increases GT toxicity, particularly in Δ*mtrA* mutants. This underscores that an intact mitochondrial function is crucial for GT resistance in *A. fumigatus* and implicates MtrA (and to a lesser degree NmrC) in protecting mitochondrial processes during GT stress.

### MtrA and NmrC are not important for virulence in a chemotherapeutic murine model of aspergillosis

To determine the impact of MtrA and NmrC deletion strains on virulence in a mammalian model, we used a chemotherapeutic murine model of infection. *A. fumigatus* Δ*mtrA* and Δ*nmrC* mutants have the same virulence as the wild-type in a chemotherapeutic murine model, as measured by animal survival (see Fig. S4a and b at https://doi.org/10.6084/m9.figshare.29825336.v3). Our results indicate that although MtrA and NmrC are important for GT production and self-defense, they are not involved in virulence in a chemotherapeutic murine model.

## DISCUSSION

*A. fumigatus* GT production involves and impacts several metabolic pathways, such as oxidative stress, sulfur assimilation, glutathione production, methyl group transfer through S-adenosyl-L-methionine, and iron and zinc metabolism (4). By using a comparative transcriptional approach, we have investigated which genes are important for coping with GT resistance and production and are dependent on the master transcriptional regulator RglT (5). Previously, we demonstrated that RglT is conserved in several *Aspergilli* and controls most of the GT BGC genes, *gliT*, and *gtmA* (5, 6). Here, we extended these studies by examining five genes that are under the transcriptional control of RglT upon GT exposure in both *A. fumigatus* and *A. nidulans. A. fumigatus* RglT influences four of these genes (*mfsD*, *mtrA*, *nmrC*, and *oxrA*) indirectly, and on *abcC1* is direct since RglT can bind to the promoter region only of *abcC1* as determined by ChIP-seq (Chromatin Immunoprecipitation coupled to DNA sequencing) (5). We constructed multiple deletion strains for all these genes in *A. fumigatus* and *A. nidulans* and extensively phenotyped them. All these null mutants are more sensitive to GT in both species, except for *A. fumigatus* Δ*oxrA* (*oxrA* encodes a putative oxidoreductase), which is more GT resistant. All the other *A. fumigatus* mutants have significantly reduced production of GT and bmGT, except for Δ*oxrA* and Δ*mtrA* (*mtrA* encodes a methyltransferase). The Δ*oxrA* and Δ*mtrA* have comparable GT production with the wild-type strain but increased bmGT production. This suggests that although GT sensitivity often correlates with reduced GT biosynthesis (e.g., in Δ*mfsD,* Δ*abcC1*, and Δ*nmrC*), it is not universally true (Δ*mtrA* remains GT-sensitive despite normal GT levels). Likewise, bmGT production can be uncoupled from GT production, as seen by increased bmGT in Δ*mtrA* and Δ*oxrA*. Thus, the mechanisms controlling bmGT (detoxification via methylation) can operate independently of those controlling GT biosynthesis.

Two of these genes encode transporters, one from the major facilitator superfamily (MFS), *mfsD*, and another, *abcC1*, a member of the ATP-binding cassette (ABC) superfamily. We were not able to assign any phenotype to the *mfsD* null mutant under the conditions tested, but *A. fumigatus* Δ*abcC1* is more sensitive to diamide, a thiol-oxidizing agent that rapidly reduces GSH (27), whereas *A. nidulans* Δ*abcC1* is more resistant. Several reactions in GT production and detoxification are mediated via GSH, including the chemical reduction of GT to dtGT and the enzymatic activity of GliG that needs 2 moles of GSH to form a bis-glutathionylated biosynthetic intermediate, which impacts the methionine/cysteine cycle (4). It is tempting to speculate that since *A. nidulans* is not a GT producer and does not need GSH for GT production, but only for GT defense, and *A. fumigatus* needs GSH for both processes, *A. nidulans* AbcC1 impacts only GT defense, whereas *A. fumigatus* AbcC1 impacts both processes. There may be more available free GSH for *A. nidulans* than for *A. fumigatus* to be used during global metabolism. This could help to explain why *A. fumigatus* is more sensitive to diamide, and *A. nidulans* is more resistant. However, in both species, we do not know the specific functions of AbcC1 and MfsD.

We also identified *mtrA,* which encodes a putative methyltransferase that affects the SAM and SAH homeostasis upon GT exposure, suggesting that MtrA could be dependent on S-Adenosyl-L-methionine (SAM), a naturally occurring trialkyl sulfonium molecule that is typically associated with biological methyl transfer reactions. The *mtrA* genes encode a methyltransferase apparently without any *S. cerevisiae* homologs. *A. fumigatus* and *A. nidulans* MtrA homologs are members of the methyltransferase family and have an S-adenosyl-L-methionine-dependent methyltransferase domain (PTHR43591:SF108; https://pantherdb.org/panther/). During the GT detoxification process, GliT removes excess GT by reducing GT to dithiol-GT (dtGT), and dtGT is removed by its conversion by GtmA into bisdethiobis(methylthio)-gliotoxin (bmGT). GtmA conversion to bmGT attenuates GT production post-biosynthetically (4). GT attenuation and removal of dtGT through methylation by GtmA spend 2 moles of SAM, and the product SAH has to be converted again to SAM via the methyl/methionine cycle (18, 28, 29). We have no evidence showing additional methylation of GT, dtGT, and bmGT, suggesting that MtrA is transferring methyl groups to other molecules that are participating in the GT self-defense.

NmrC is a homolog of a group of proteins that includes those containing the NmrA domain. NmrA acts as a negative transcriptional regulator in various fungi and plays a role in the post-translational modulation of the GATA-type transcription factor AreA (30–32). It has been shown that NmrA consists of two domains, including a Rossmann fold. NmrA is closely related to the SDR (short-chain dehydrogenases/reductases) family, with the strongest relationship to UDP-galactose 4-epimerase (33). Unlike typical proteins in its class, NmrA does not possess the canonical GXXGXXG NAD-binding motif and contains modified residues at the catalytic triad, specifically a methionine instead of the critical tyrosine residue. Although NmrA may bind nucleotides, it does not display any dehydrogenase activity and lacks the most active site residues found in the SDR family. However, it features a NAD(P)-binding motif similar to that of the extended SDR family, GXXGXXG (33). Although NAD binds to NmrA, it is unlikely that NmrA functions as an active dehydrogenase. Instead, the nucleotide-binding may serve a regulatory purpose (33). The SDR family may have evolved for various roles, including non-enzymatic control functions such as transcriptional regulation (33). There are four homologs of the NmrA-like domain-containing protein in *A. fumigatus* A1163 and seven homologs in *A. nidulans* A4. We previously characterized one of these *A. nidulans* homologs, NmrB (34). Deletion of this gene resulted in increased susceptibility to oxidative stress caused by hydrogen peroxide and menadione (34).

Interestingly, *A. fumigatus* Δ*mtrA* and Δ*nmrC* have similar phenotypes, such as resistance to oxidative stressing agents like allyl alcohol, menadione, t-butyl hydroperoxide, and diamide, and more susceptibility to iron starvation. *A. nidulans* and *A. fumigatus* Δ*mtrA* and Δ*nmrC* mutants have different phenotypes, that is, *A. nidulans* is more sensitive to oxidative stressing agents and resistant to diamide. Once again, these phenotypic differences could reflect the impact of GT on gene regulation in both species, and this remains to be investigated. Transcriptional profiling and String analysis showed that MtrA and NmrC interact with mitochondrial proteins and impact mitochondrial function, although they are not mitochondrially localized (TargetP 2.0 prediction). We validated their influence on mitochondrial function through the use of mitochondrial inhibitors. Curiously, the alternative oxidase gene is also important for GT susceptibility, suggesting that alternative mitochondrial pathways also play a role in GT self-protection. Moreover, exposure of *A. fumigatus* germlings to GT increases mitochondrial fragmentation, indicating that GT’s fungicidal effect involves mitochondrial dysfunction characteristic of an apoptosis-like cell death. However, this cell death does not seem to be dependent on caspases. The first systematic screening for mechanisms of GT resistance or tolerance was performed in GT non-producer *S. cerevisiae* by using deletion libraries (35). Interestingly, this screening highlighted the GT multi-target nature since mutants with increased GT resistance were associated with the general metabolism, mitochondrial function, DNA damage repair, and vesicular transport. In contrast, mutants with increased GT sensitivity were linked to transsulfuration and mitochondrial functions (35). More specifically, mutants with increased GT resistance were linked to RTG2 (a sensor of mitochondrial dysfunction) and GT-increased sensitivity to MEF2 (mitochondrial elongation factor involved in translational elongation) (35). Several studies indicate that GT triggers excessive production of reactive oxygen species and disrupts mitochondrial membrane potential. This disruption leads to the activation of both endogenous and exogenous apoptotic pathways in mammalian cells (36–42).

Our work identifies genetic determinants important for mitochondrial function upon GT exposure, adding a new layer to the *A. fumigatus* GT self-protection. Interestingly, these genes are present and functional not only in the GT producer *A. fumigatus* but also in the GT non-producer *A. nidulans*, strongly indicating that both GT producers and non-producers have to deal with the damage caused by GT to mitochondrial function. In our earlier study, we demonstrated that both RglT and GliT proteins are more frequently present in Eurotiomycetes compared to Sordariomycetes (5). Using phylogenetically informed model analyses to examine the distribution patterns of RglT and GliT, we found that the presence of RglT is statistically dependent on the occurrence of GliT, whereas the reverse relationship does not hold (5). This observation supports an evolutionary model where the GliT-mediated resistance mechanism is ancestral, and the regulatory role of RglT over GliT emerged later in evolution. However, it remains unclear whether mitochondrial protection mechanisms evolved before the biosynthesis of GT. Targeting these mitochondrial protection pathways might be a novel strategy to enhance antifungal efficacy or understand fungal interactions.

## MATERIALS AND METHODS

### Strains and media

All strains used in this study are listed in Table S3 (https://doi.org/10.6084/m9.figshare.29825336.v3). Conidia of *A. fumigatus* and *A. nidulans* were grown on minimal media (MM) [1% (wt/vol) glucose, nitrate salts, trace elements, pH 6.5]. Solid MM was the same as described above, with the addition of 2% (wt/vol) agar. Depending on the auxotrophy of the strain, uridine (1.2 g/L), uracil (1.2 g/L), or pyridoxine (0.005 mg/µL) was added. Trace elements, vitamins, and nitrate salt compositions were as described previously (43). Gliotoxin production was induced by growing the strains in Czapek-Dox (http://himedialabs.com/TD/M076.pdf) broth. For phenotypic characterization, plates were inoculated with 10^4^ spores per strain and left to grow for 120 h on MM-supplemented Allyl alcohol, menadione, t-butyl hydroperoxide, diamide, FeSO_4_ 200 µM, BPS + Ferrozine, H_2_O_2_. Strains were grown at 37°C except for growth on an allyl alcohol-containing solid medium, which was carried out at 30°C. Radial growth experiments were expressed as ratios, dividing the colony radial diameter of growth in the stress condition by the colony radial diameter in the control (no stress) condition.

### Generation of *A. nidulans* and *A. fumigatus* mutants

All gene replacement cassettes were constructed by *in vivo* recombination in *S. cerevisiae*, as previously described by (44). For the construction of *A. nidulans* and *A. fumigatus* null mutants, approximately 1.0 kb from each 5-UTR and 3-UTR flanking region of the targeted ORF regions were selected for primer design (see Table S4 at https://doi.org/10.6084/m9.figshare.29825336.v3). The primers gene_pRS426_5UTR_fw and gene_pRS426_3UTR_rv contained a short homologous sequence to the MCS of the plasmid pRS426. Both the 5- and 3-UTR fragments were PCR-amplified from the genomic DNA of *A. nidulans* AGB551 or *A. fumigatus* A1163 strains. The *pyrG* gene, placed within the cassette as a prototrophic marker, was amplified from the pCDA21 plasmid (45). The cassette was PCR-amplified from these plasmids utilizing TaKaRa Ex Taq™ DNA Polymerase (Clontech Takara Bio) and used for *A. nidulans* and *A. fumigatus* transformation. Southern blot analysis was performed to confirm the deletions (see Fig. S3 at https://doi.org/10.6084/m9.figshare.29825336.v3).

### The mito::GFP strain

The strain was constructed as described by (46). *A. fumigatus* candidates were selected and purified through three rounds of growth on plates. The gDNA of the mutants was extracted, and the construction insertion was confirmed by PCR. Primer sequences are listed in Table S4 (https://doi.org/10.6084/m9.figshare.29825336.v3).

### Gliotoxin and bmGT extraction and analysis by high-performance liquid chromatography (HPLC)

The *A. fumigatus* strains A1163, Δ*mtrA*, Δ*gtmA*, Δ*mtrA* Δ*gtmA*, Δ*abcC1*, Δ*mfsD*, and Δ*nmrC* were grown in Czapek-Dox broth for 72 h. For extraction, the cultures were submitted to a liquid-liquid partition with 15 mL, 300 mL, and 300 µL of chloroform, respectively, three times. Organic fractions were washed with a saturated solution of NaCl and dried with anhydrous Na_2_SO_4_. The suspensions were filtered and concentrated under a vacuum. The instrumentation for the HPLC used was the Shimadzu Nexera XR LC-20AD (Kyoto, Japan) chromatography model, consisting of CBM-20A control, SPD-M20A DAD, and ELSD-LTII evaporative light scattering detectors, Nexera SIL-20A auto-injector, CTO-20A oven, using reversed-phase C18 column (Ascentis, 2.7 µm, 100 × 4.6 mm, 35°C) with gradient of aqueous (0.1% acetic acid) and acetonitrile (10% to 100% of acetonitrile) for 35 min. LabSolutions software (Shimadzu Corporation, Kyoto, Japan) was used for data acquisition and analysis.

### Determination of cellular SAM and SAH levels

*A. fumigatus* strains were grown in liquid MM for 21 h at 37 ͦC and then exposed to 5 µg/mL GT for 3 h. Mycelia were ground in liquid nitrogen, resuspended in cold PBS, and centrifuged at 10,000  ×  *g* for 15 min at 4°C. The supernatants were collected and stored on ice. The determination of cellular SAM and SAH levels using the kit from Cell Biolabs [S-Adenosylmethionine (SAM) and S-adenosylhomocysteine (SAH) ELISA Combo Kit, MET-5151-C] was performed according to the manufacturer’s instructions. The endpoint reaction was detected using a Synergy-HT microplate reader (Bio-Tek) at 450 nm. SAM and SAH concentrations were normalized by wet mycelia weight.

### Total glutathione concentrations

Total glutathione levels were determined with the GSH  + GSSG/GSH Assay Kit (Colorimetric) (Abcam, ab239709). Mycelia were ground to a fine powder in liquid nitrogen and re-suspended in Glutathione buffer (provided by the kit), according to the manufacturer’s instructions. The assay was based on the glutathione recycling system by 5,5'-dithiobis-(2-nitrobenzoic acid) (DTNB) and glutathione reductase. DTNB and glutathione (GSH) react to generate 2-nitro-5-thiobenzoic acid, which could be detected at 412 nm, using a Synergy-HT microplate reader (Bio-Tek). Glutathione concentrations were calculated from a GSH standard curve and were normalized by fungal dry weight.

### Animal survival curves and fungal burden

Inbred female mice (BALB/c strain; body weight, 20–22 g; age of 8–9 weeks) were housed in vented cages containing five animals. Cages are well-ventilated, softly lit, and subjected to a 12:12 light-dark cycle. The relative humidity was kept at 40% to 60%. Mouse rooms and cages were kept at a temperature range of 22°C. Mice were immunosuppressed with cyclophosphamide (150 mg/kg of body weight), which was administered intraperitoneally on days −4,−1, and 2 before and post-infection (infection day is “day 0”). Hydrocortisone acetate (200 mg/kg body weight) was injected subcutaneously on day −3. Mice (10 mice per group, two repetitions) were anesthetized by halothane inhalation and infected by intranasal instillation of 20 µL containing 1.0 × 10^5^ conidia of *A. fumigatus* wild-type or mutant strains. The viability of the administered inoculum was determined by incubating different serial dilutions of the conidia used in both repetitions on MM medium at 37°C. As a negative control, a group of 10 mice received PBS only. Animals were sacrificed 15 days post-infection or if moribund.

The principles that guide our studies are based on the Declaration of Animal Rights ratified by UNESCO on January 27, 1978, in its 8th and 14th articles. All protocols adopted in this study were approved by the local ethics committee for animal experiments from the University of São Paulo, Campus of Ribeirão Preto (Permit Number: 23.1.547.60.8; Characterization of virulence and immunopathogenicity of *Aspergillus* spp in the murine model). Groups of five animals were housed in individually ventilated cages and were cared for in strict accordance with the principles outlined by the Brazilian College of Animal Experimentation (COBEA) and Guiding Principles for Research Involving Animals and Human Beings, American Physiological Society. All efforts were made to minimize suffering. Animals were clinically monitored at least twice daily and humanely sacrificed if moribund (defined by lethargy, dyspnea, hypothermia, and weight loss). All stressed animals were sacrificed by cervical dislocation.

### Fluorescence microscopy

A total of 10^5^ spores of *A. fumigatus* strain with mitochondria constitutively expressing GFP (to assess mitochondrial fragmentation) or *A. fumigatus* conidia containing the histone h2A::RFP were inoculated on coverslips in 4 mL of MM for 16 h at 37°C. The coverslips with adherent germlings were left untreated or treated with gliotoxin 5 µg/mL for 3 h or H_2_O_2_ 10 mM for 30 min. After each period, the cells were stained with Hoechst 33342 nucleic acid dye (20 mg/mL; Molecular Probes, Eugene). The coverslips were visualized on the Observer Z1 fluorescence microscope using 100× oil immersion lens objectives. Differential interference contrast (DIC) and fluorescent images were captured with an AxioCam camera (Carl Zeiss) and processed using the AxioVision software (version 4.8). The wavelength excitation for h2A::RFP was 572/25 nm, and the emission wavelength was 629/62 nm; for Hoechst staining, the excitation wavelength was 365 nm, and the emission wavelength was 420–470 nm; and for GFP, the wavelength excitation was 450–490 nm, and the emission wavelength was 500–550 nm.

### RNA extraction and RNA-sequencing

All experiments were carried out in biological triplicate, and conidia (10^7^) were inoculated in liquid MM. Gliotoxin was added for 3 h to the culture medium to a final concentration of 5 µg/mL after strains were grown for 21 h in MM. For total RNA isolation, mycelia were ground in liquid nitrogen, and total RNA was extracted using TRIzol (Invitrogen), treated with RQ1 RNase-free DNase I (Promega), and purified using the RNAeasy kit (Qiagen) according to the manufacturer’s instructions. RNA was quantified using a NanoDrop and Qubit fluorometer and analyzed using an Agilent 2100 Bioanalyzer system to assess the integrity of the RNA. All RNA had an RNA integrity number (RIN) between 8.0 and 10 (Thermo Scientific) according to the manufacturer’s protocol. Total RNA samples were submitted to the Novogene company (Beijing, China) to proceed with the library preparation. A strand-specific library was prepared using an Illumina TruSeq strand-specific RNA sample preparation system. The DNA library of 250–300  bp insert size was constructed and sequenced using an Illumina NovaSeq 6000 platform with a 150 bp paired-end sequencing strategy. The 36 libraries obtained from 101.26 million to 207.66 million high-quality reads, with 95.35%–97.18% clean bases and less than 0.03% base error rate for all samples were achieved. The reference genome and annotations used in the analysis were from the strain A. fumigatus Af293 (NCBI genome accession GCA_000002655.1).

### Statistical analysis

Grouped column plots with standard deviation error bars were used for representations of data. Graphics, buildings, and statistical analyses were performed using the GraphPad Prism 8 (GraphPad Software). For comparisons with data from the wild-type strain or control conditions, the one-tailed paired *t*-test and one-way or two-way analysis of variance (ANOVA) were performed.

## Data Availability

All the data are available as supplementary tables and figures. RNA-sequencing data was data were deposited at NCBI under the accession number: PRJNA1240253 ID: 1240253.
